# A lightweight region of interest-level adjudication framework with hard-negative mining and confidence-aware fusion for pediatric fracture detection

**DOI:** 10.3389/frai.2026.1807189

**Published:** 2026-05-04

**Authors:** C. V. Aravinda, Noushath Shaffi, Vimbi Viswan, Adham Al-Rahbi, Choy Ker Woon, Yassine Bouchareb, Srinivasa Rao Sirasanagandla

**Affiliations:** 1 Department of Computing and Electronics Engineering, Middle East College (Affiliated With Coventry University), Muscat, Oman; 2 Department of Computer Science, College of Science, Sultan Qaboos University, Muscat, Oman; 3 College of Computing and Information Science, University of Technology and Applied Sciences, Sohar, Oman; 4 Anatomical Pathology Program, Oman Medical Specialty Board, Muscat, Oman; 5 Department of Anatomy, Faculty of Medicine, Universiti Teknologi MARA, Sungai Buloh, Malaysia; 6 Department of Radiology and Molecular Imaging, College of Medicine and Health Sciences, Sultan Qaboos University, Muscat, Oman; 7 Department of Human and Clinical Anatomy, College of Medicine and Health Sciences, Sultan Qaboos University, Muscat, Oman

**Keywords:** confidence calibration, deep learning, explainable AI, fracture detection, hard-negative mining, medical image analysis, region of interest adjudication

## Abstract

Accurate detection of pediatric fractures in radiographs remains challenging due to subtle visual cues and the high prevalence of false-positive detections produced by automated systems. To address this limitation, we propose a lightweight region-of-interest (Region of Interest) adjudication framework that operates as a second-stage verification module to refine detector-generated candidates. The proposed framework integrates iterative hard-negative mining with confidence-aware score fusion to suppress anatomically confounding regions such as growth plates and overlapping structures. Unlike end-to-end detection approaches, the method is designed to function as a modular post-detection refinement stage, enabling improved decision reliability without modifying the underlying detector architecture. Each candidate Region of Interest is evaluated using a compact adjudication network conditioned on detector confidence, and final predictions are obtained through a calibrated fusion strategy. The framework is evaluated on the publicly available GRAZPEDWRI-DX pediatric radiograph dataset using patient-level disjoint training, validation, and held-out test splits to ensure unbiased performance estimation. Experimental results demonstrate that the proposed approach reduces false-positive detections while maintaining high sensitivity. At the selected operating point, the method achieves an F1-score of 0.88 and mAP@0.5 of 0.887, outperforming the detector-only baseline under identical evaluation conditions. In addition, gradient-based activation mapping (Grad-CAM) is employed to provide Region of Interest-level visual explanations, supporting interpretability of adjudication decisions. The proposed framework maintains low computational overhead, making it suitable for integration into real-world clinical workflows as a decision-support component.

## 1 Introduction

Musculoskeletal injuries, particularly long-bone fractures, represent one of the most common causes of emergency radiographic examinations worldwide and constitute a major public health burden (Sharma, 2023). These injuries may arise from traumatic events or underlying bone pathologies and can significantly affect patient mobility, quality of life, and long-term clinical outcomes (Bliuc et al., 2009; Pour and Berretti, 2025). The incidence of fractures is influenced by multiple factors, including age-related bone degeneration, osteoporosis, malignancies, and accidental trauma, with pediatric populations presenting additional anatomical variability due to skeletal immaturity.

Accurate and timely identification of fractures is essential to prevent complications such as malunion, neurovascular injury, and delayed rehabilitation (Han et al., 2025). However, fracture detection in radiographs remains challenging even for experienced radiologists, particularly in high-volume clinical environments. Prior studies have reported non-negligible diagnostic discrepancies, with missed or over-called findings occurring in routine musculoskeletal imaging (Huhtanen et al., 2023; Hallas and Ellingsen, 2006). Subtle fracture patterns, overlapping anatomical structures, and growth plates in pediatric patients further increase diagnostic complexity (Lindsey et al., 2018; Dandár et al., 2025).

In recent years, artificial intelligence (AI)-based computer-aided diagnosis systems have emerged as valuable decision-support tools in musculoskeletal radiology. Deep learning models based on convolutional neural networks, transformers, and object-detection architectures have demonstrated promising performance in automated fracture detection and localization (Hong et al., 2020; Alam et al., 2024; Nazim et al., 2025). Despite these advances, several challenges limit their practical deployment. Many high-recall detectors generate a substantial number of false-positive regions of interest (Region of Interests), which can overwhelm clinicians and reduce trust in automated predictions. Additionally, most detection models provide limited interpretability, offering minimal insight into the visual evidence underlying their decisions (Tjoa and Guan, 2020; Kinger and Kulkarni, 2024).

Another underexplored limitation is the scarcity of challenging non-fracture examples that closely resemble true fractures in appearance. Such hard negatives commonly arise from growth plates, overlapping bones, or imaging artifacts, particularly in pediatric radiographs. Insufficient exposure to these patterns during training can negatively affect model calibration and generalization (Oakden-Rayner et al., 2020; Guo et al., 2017). While existing studies predominantly focus on end-to-end detection performance, relatively little attention has been given to post-detection refinement mechanisms that verify or reject detector-generated candidates.

Motivated by these observations, this work focuses on Region of Interest-level adjudication as a complementary stage to existing fracture detection pipelines. Rather than replacing detector models, we propose a lightweight adjudication framework that refines candidate Region of Interests by suppressing anatomically confounding false positives and providing interpretable decision cues. The framework integrates iterative hard-negative mining to progressively strengthen discrimination against challenging non-fracture regions and employs confidence-aware score fusion to stabilize Region of Interest-level predictions. To support transparency, gradient-based activation visualizations are used to highlight regions contributing to adjudication decisions.

The main contributions of this work are summarized as follows:

- A lightweight Region of Interest-level adjudication framework is presented to refine detector-generated fracture candidates by suppressing anatomically confounding false positives in pediatric radiographs.
- An iterative hard-negative mining strategy is introduced to systematically incorporate challenging non-fracture Region of Interests during training, improving robustness and generalization.
- A confidence-aware score fusion mechanism is developed to integrate detector confidence with adjudicator predictions, enabling stable and interpretable Region of Interest-level decisions.
- Qualitative explainability analyses based on gradient-driven activation visualizations are provided to support transparency of the adjudication process.

By addressing false-positive suppression, calibration stability, and interpretability within a single modular component, this work aims to advance clinically transparent and reproducible AI-assisted fracture analysis. The remainder of this paper is organized as follows. Section 2 reviews related work in fracture detection, lightweight architectures, explainable AI, and hard-negative mining. Section 3 presents the proposed Region of Interest adjudication framework, including the model architecture, confidence fusion strategy, and training procedure. Section 4 describes the experimental setup, dataset, evaluation metrics, and results. Section 5 concludes the paper and outlines directions for future work.

## 2 Literature review

This literature review synthesizes prior research across five critical domains relevant to quantum-inspired hard-negative-mined Region of Interest adjudication for bone fracture verification: AI-based fracture detection and localization, lightweight architectures for clinical deployment, explainable AI techniques, hard-negative mining and model calibration strategies, and quantum-inspired learning approaches. The review identifies key technological advances, performance benchmarks, and research gaps that motivate the development of QHRA as a comprehensive solution for clinical bone fracture verification systems.

### 2.1 AI-based fracture detection and localization

Traditional fracture detection has evolved significantly with the integration of artificial intelligence, particularly deep learning methods. Early CNN-based approaches demonstrated promising results, with models like ResNet-50 achieving accuracy rates of 96.2% for fracture classification (Sudha et al., 2025). Contemporary research has increasingly focused on object detection frameworks, with YOLO variants showing substantial improvements in fracture localization. Recent studies utilizing YOLOv8 and YOLOv9 on the GRAZPEDWRI-DX dataset have achieved mean average precision (mAP) values ranging from 63.6% to 66.3% (Ju and Cai, 2023; Ju and Chen, 2024). Vision Transformers have also emerged as competitive alternatives, with some architectures demonstrating superior contextual understanding through self-attention mechanisms (Guan et al., 2024).

Multi-scale feature fusion approaches have shown particular promise for complex fracture detection scenarios. The Multi-Level Feature Fusion Network (MLFNet) achieved 99.89% accuracy on the Bone Fracture Multi-Region X-ray dataset by integrating both low-level and high-level image features (Abd El-Ghany et al., 2025).

Contemporary studies report classification accuracies exceeding 99% on specialized datasets, though these results often reflect controlled experimental conditions rather than clinical deployment scenarios (Sushma et al., 2025).

Despite these advances, significant limitations persist in current AI-based fracture detection systems. High false positive rates remain problematic in clinical settings, with studies reporting specificity challenges when deployed on diverse patient populations (Kutbi, 2024). Most importantly, existing systems lack robust Region of Interest-level verification mechanisms, focusing primarily on initial detection rather than confirmatory adjudication of suspected fracture regions. This gap necessitates the development of specialized adjudicator systems that can provide secondary validation of detected fracture candidates.

### 2.2 Lightweight and efficient architectures for clinical AI

The deployment of AI systems in clinical environments requires architectures that balance diagnostic accuracy with computational efficiency. MobileNet variants have demonstrated particular success in medical imaging applications, with MobileNetV3 achieving competitive performance while maintaining compact model sizes suitable for edge deployment (Kubakisa et al., 2025). EfficientNet architectures have similarly shown promise, with the EfficientNet-B3 model achieving 94.7% accuracy in medical item classification while requiring significantly fewer parameters than traditional CNN architectures (Riaz et al., 2025).

Recent developments in lightweight CNN design have focused on depth-wise separable convolutions and attention mechanisms. The incorporation of Convolutional Block Attention Modules (CBAM) into lightweight architectures has shown improvements in medical image analysis tasks, with models achieving 99.98% training accuracy while maintaining computational efficiency (Sumon et al., 2025). Ghost convolutions have emerged as another promising approach, generating equivalent feature representations with reduced linear operations, leading to 68.7% size reductions compared to standard architectures (Ferdi, 2024).

Specialized lightweight architectures for medical applications have demonstrated superior performance in resource-constrained environments. The SANS-CNN model, incorporating an EfficientNet encoder, achieved 84.2% accuracy for astronaut medical imaging with minimal computational overhead (Kamran et al., 2024). Multi-scale fusion lightweight networks (MFLUnet) have shown particular promise for medical image segmentation, effectively extracting both local and global features while maintaining low computational complexity (Cao et al., 2024). However, despite these advances, few lightweight architectures are specifically designed as Region of Interest adjudicators, representing a significant gap in clinical AI deployment strategies.

### 2.3 Explainable AI (XAI) and clinical interpretability

The clinical adoption of AI systems fundamentally depends on their interpretability and trustworthiness. Gradient-weighted Class Activation Mapping (Grad-CAM) has emerged as a standard visualization technique, providing intuitive heatmaps that highlight decision-relevant regions in medical images (Barra et al., 2024). SHAP (SHapley Additive exPlanations) methods have demonstrated superior fidelity in medical imaging applications, achieving 0.81 fidelity scores compared to 0.54 for Grad-CAM across multiple modalities (Singh et al., 2025). LIME (Local Interpretable Model-agnostic Explanations) has shown particular utility in identifying when models rely on irrelevant background features, though its computational overhead limits real-time applications (Jadhav et al., 2025).

Ensemble XAI approaches have shown promise in clinical settings, with combined SHAP and Grad-CAM++ methods achieving higher radiologist trust scores in pneumonia classification tasks (Zou et al., 2022). Recent studies demonstrate that XAI techniques significantly improve diagnostic accuracy when integrated with clinical workflows, with physicians showing 87% fracture detection sensitivity when assisted by CNN models with integrated explainability features (Stonko et al., 2021).

A critical limitation in current XAI applications is the lack of localized reasoning for Region of Interest-level decisions. Most explainability methods provide global image-level explanations rather than region-specific justifications, limiting their utility for confirmatory diagnosis tasks. Additionally, the clinical validation of XAI outputs remains insufficient, with limited studies demonstrating improved patient outcomes through explainable AI integration. These gaps highlight the need for specialized explainability frameworks designed for Region of Interest adjudication tasks.

### 2.4 Hard-negative mining and model calibration in medical imaging

Hard-negative mining techniques have shown significant potential for improving model performance in imbalanced medical imaging datasets. Online Hard Example Mining (OHEM) has demonstrated effectiveness in lesion detection tasks, with studies reporting improved precision and recall through iterative hard-negative selection (Cai et al., 2020). Focal loss modifications have addressed class imbalance issues in medical segmentation, with region-related focal loss achieving superior performance in brain tumor MRI segmentation tasks (Li et al., 2023). Mixed-order minibatch sampling strategies have further enhanced training effectiveness by optimizing sample utilization across diverse quality images (Li et al., 2025).

Model calibration techniques have become increasingly important for clinical deployment, where prediction confidence directly impacts diagnostic decisions. Temperature scaling has shown consistent improvements in medical AI calibration, though integration with hard-negative mining strategies remains limited. Recent work on uncertainty-aware consistency learning has demonstrated improved performance in semi-supervised medical image segmentation, with dynamic weighting based on prediction uncertainty (Assefa et al., 2025). Contrastive learning approaches incorporating hard-negative mining have shown promise in medical image classification, particularly for low-quality image scenarios (Hou et al., 2024).

Despite these advances, the integration of hard-negative mining with model calibration remains underexplored in clinical applications. Most studies focus on either mining strategies or calibration techniques in isolation, missing opportunities for synergistic improvements. Furthermore, few frameworks specifically address the unique requirements of Region of Interest adjudication, where the model must distinguish between subtle variations in candidate regions rather than performing primary detection tasks.

### 2.5 Quantum-inspired and hybrid learning in medical AI

Quantum-inspired neural networks represent an emerging paradigm in medical AI, leveraging quantum computing principles to enhance classical machine learning approaches. Quantum Convolutional Neural Networks (QCNNs) have demonstrated competitive performance in medical image classification, with recent studies achieving 99.67% accuracy in brain tumor detection while maintaining computational efficiency (Khan et al., 2024). Hybrid quantum-classical architectures have shown particular promise, with quantum feature-encoding circuits integrated with classical neural networks achieving superior performance in resource-constrained environments (Ajlouni et al., 2023).

Recent developments in quantum-inspired learning have focused on complex convolutional operations and parameterized quantum circuits. The Quantum-inspired Complex Convolutional Neural Network (QICCNN) achieved 99.89% accuracy in brain tumor classification by incorporating quantum circuit-based feature mapping (Subhashini and Thangakumar, 2024). Medical image segmentation applications have benefited from quantum-inspired neural architecture search (SegQNAS), demonstrating improved performance over traditional approaches in spleen and prostate segmentation tasks (Carlos et al., 2023). Quantum Support Vector Machines integrated with classical preprocessing pipelines have shown effectiveness in neuRegion of Interestmaging applications, particularly for high-dimensional data scenarios (Chen et al., 2025).

However, significant limitations exist in current quantum-inspired medical AI applications. Most studies focus on classification tasks rather than specialized applications like Region of Interest adjudication. The integration of quantum-inspired approaches with explainability requirements remains largely unexplored, limiting their clinical applicability. Furthermore, no prior framework has demonstrated the application of quantum-inspired learning within a clinically explainable adjudication setting, representing a significant research gap that QHRA aims to address.

The literature review reveals several critical gaps that motivate the development of QHRA. First, while fracture detection systems achieve high accuracy rates, they lack specialized Region of Interest-level adjudication capabilities for confirmatory diagnosis. Second, lightweight architectures suitable for clinical deployment have not been specifically optimized for adjudication tasks, focusing primarily on primary detection rather than secondary verification. Third, explainable AI methods provide limited localized reasoning for Region of Interest-specific decisions, with most approaches offering global rather than region-specific explanations.

Fourth, the integration of hard-negative mining with model calibration remains underexplored in medical imaging, particularly for adjudication scenarios where subtle distinctions between candidate regions are critical. Finally, quantum-inspired learning approaches have not been applied to clinically explainable adjudication settings, missing opportunities to leverage quantum computing principles for enhanced diagnostic verification. QHRA addresses these gaps through: (i) specialized lightweight adjudication architecture, (ii) localized explainable overlays for Region of Interest-specific reasoning, (iii) integrated calibration with hard-negative mining for improved reliability, and (iv) quantum-inspired reasoning mechanisms within a clinically interpretable framework.

## 3 Proposed methodology

**Figure 1:**
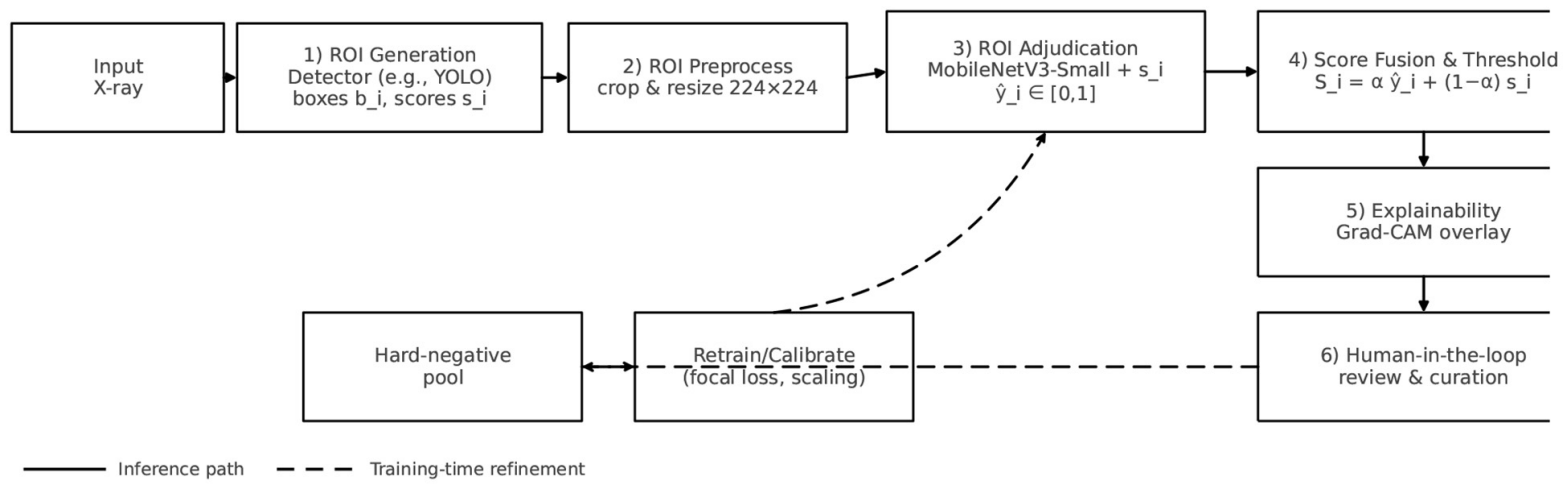
Quantum hard-negative-mined ROI adjudicator workflow. Detector proposals are verified by a lightweight adjudicator, scores are fused and thresholded, and Grad-CAM explains the decision. During training, clinician feedback, and convincing false alarms feed a hard-negative pool for iterative improvement.

The proposed Hard-Negative-mined Region of Interest Adjudicator (Quantum Hard-Negative-mined ROI Adjudicator) is designed as a lightweight and interpretable verification layer that operates downstream of high-sensitivity fracture detectors such as YOLO. Rather than performing fracture detection independently, Quantum Hard-Negative-mined ROI Adjudicator refines detector-generated regions of interest (Region of Interests) by suppressing anatomically confounding false positives, calibrating prediction confidence, and providing visual explanations to support image interpretation. The overall workflow, illustrated in Figure 1, consists of Region of Interest generation, preprocessing, adjudication, confidence fusion, hard-negative mining, and explainability.

### 3.1 Problem formulation

Automatic fracture detection in radiographs can be formulated as a two-stage decision process: (i) a primary detector generates high-recall regions of interest (Region of Interests) that may contain fractures, and (ii) a secondary adjudication stage verifies the validity of each proposed region. While modern detectors such as YOLO efficiently localize candidate fracture regions, they frequently produce redundant or spurious proposals, particularly in anatomically complex pediatric radiographs, motivating the need for an intelligent Region of Interest-level verification mechanism.

Formally, let a radiograph image be represented as *I* ∈ ℝ^*H*×*W*×*C*^, where *H* and *W* denote the image height and width, and *C* denotes the number of channels (with *C* = 1 for grayscale radiographs). A detector $D_{\theta_{d}}$ produces a set of candidate bounding boxes


$$B=\left\{\left(b_{i},s_{i}\right)|i=1,2,\ldots ,N\right\},$$

where $b_{i}\in {\mathbb{R}}^{4}$ denotes the coordinates of the *i*^th^ Region of Interest and *s*_*i*_ ∈ [0, 1] represents the associated detector confidence score.

The objective of the proposed Region of Interest adjudication framework is to learn a mapping


$$A_{\theta_{a}}:\;\left(I_{b_{i}},s_{i}\right)\to {\hat{y}}_{i},$$

where *I*_*b*_*i*__ denotes the image patch corresponding to Region of Interest *b*_*i*_, and ŷ_*i*_ ∈ [0, 1] represents the predicted probability of a true fracture within that region. These predictions are subsequently combined with detector confidences through a confidence-aware fusion strategy to obtain calibrated decision scores that determine whether each Region of Interest is accepted or rejected.

The primary challenges addressed in this formulation include: (i) distinguishing subtle fracture cues from visually similar normal anatomical patterns, (ii) reducing false-positive detections without sacrificing sensitivity, (iii) learning effectively from hard-negative samples that closely resemble true fractures, and (iv) producing interpretable and confidence-calibrated outputs that can assist clinical image interpretation. Overall, the goal is to design a lightweight, interpretable, and data-efficient Region of Interest adjudication framework that bridges the gap between automated detection and reliable post-detection verification.

### 3.2 Region of interest generation

Region of Interest proposals are generated using a YOLOv8 object detection model configured for high-sensitivity fracture localization. The detector is initialized with pre-trained weights and fine-tuned on the GRAZPEDWRI-DX dataset using the same patient-level training split described in Section 4.

During inference, radiographs are resized to 640 × 640 pixels and processed to produce candidate bounding boxes along with associated confidence scores. A low confidence threshold (e.g., 0.25) is used to prioritize recall, and non-maximum suppression (NMS) with an IoU threshold of 0.5 is applied to remove redundant proposals.

This configuration intentionally favors high sensitivity to ensure that potential fracture regions are not missed, with the understanding that false-positive proposals are subsequently refined by the Region of Interest adjudication stage.

### 3.3 Region of interest preprocessing

Each candidate Region of Interest *I*_*b*_*i*__ is cropped from the original radiograph, resized to 224 × 224 pixels, and intensity-normalized using ImageNet statistics. During training, moderate data augmentations including random rotation, brightness variation, and affine transformations are applied to improve generalization and robustness to acquisition variability. The adjudication network is based on the MobileNetV3-Small architecture, chosen for its efficiency and suitability for lightweight deployment (Howard et al., 2019).

### 3.4 Region of interest adjudication network

Each preprocessed Region of Interest *I*_*b*_*i*__, together with its detector confidence *s*_*i*_, is passed to a compact adjudication network $A_{\theta_{a}}$ based on a **MobileNetV3-Small** backbone. The network extracts a feature embedding *f*(*I*_*b*_*i*__), which is concatenated with the detector confidence prior to classification:


$$z_{i}=\left[f\left(I_{b_{i}}\right);s_{i}\right],\;{\hat{y}}_{i}=\sigma \left(W_{2}\phi \left(W_{1}z_{i}+b_{1}\right)+b_{2}\right),$$

where ϕ(·) and σ(·) denote the ReLU and sigmoid activation functions, respectively. The output ŷ_*i*_ ∈ [0, 1] represents the adjudicator's estimated probability that the Region of Interest contains a true fracture. Conditioning the classifier on detector confidence enables the network to learn when to reinforce or correct the detector's initial assessment.

### 3.5 Confidence fusion and calibration

Final Region of Interest-level decision scores are obtained by combining the adjudicator output with the detector confidence through a convex fusion rule:


$$S_{i}=\alpha {\hat{y}}_{i}+\left(1-\alpha \right)s_{i},$$ (1)

where α ∈ [0, 1] controls the relative contribution of the adjudicator. A decision threshold τ is selected on a validation set to maximize the F1-score, yielding the final classification ỹ_*i*_ = 1 if *S*_*i*_ ≥ τ and 0 otherwise. This fusion strategy stabilizes predictions by balancing detector sensitivity with adjudicator reliability.

Equation 1 defines a convex combination of the adjudicator output ŷ_*i*_ and the detector confidence *s*_*i*_, where the parameter α controls the relative contribution of each component. This formulation balances the high sensitivity of the detector with the refinement capability of the adjudicator, resulting in more stable ROI-level predictions.

### 3.6 Hard-negative mining

To improve discrimination against anatomically ambiguous patterns, an iterative hard-negative mining strategy is employed during training. False-positive Region of Interests satisfying


$$\left(S_{i}>\tau \wedge y_{i}=0\right)$$

are collected as hard negatives:


$$N_{h}=\left\{I_{b_{i}}|S_{i}>\tau \wedge y_{i}=0\right\}.$$ (2)

The training dataset is progressively expanded as


$$D^{\prime }=P\cup N_{0}\cup N_{h},$$

where P and $N_{0}$ denote the original positive and negative samples, respectively. This curriculum-style refinement exposes the model to challenging non-fracture Region of Interests such as growth plates and overlapping bone edges, improving robustness and generalization.

### 3.7 Learning objective

Each Region of Interest is associated with a binary label *y*_*i*_ ∈ {0, 1}. The adjudicator is trained using focal loss to address class imbalance and emphasize difficult examples:


$$L_{\text{focal}}=-\frac{1}{N}\overset{N}{\underset{i=1}{\sum }}\left[{\left(1-{\hat{y}}_{i}\right)}^{\gamma }y_{i}\log{\hat{y}}_{i}+{\hat{y}}_{i}^{\gamma }\left(1-y_{i}\right)\log\left(1-{\hat{y}}_{i}\right)\right],$$ (3)

where γ > 0 controls the degree of emphasis on misclassified samples.

Equation 3 represents the focal loss used to address class imbalance and emphasize difficult samples. The parameter γ controls the degree to which misclassified examples are up-weighted, enabling the model to focus on hard-negative ROIs that are visually similar to fractures.

The adjudicator is trained using focal loss to address class imbalance and emphasize difficult examples: (Lin et al., 2017).

### 3.8 Normalized representation and feature stability

To promote stable Region of Interest representations, feature embeddings are L2-normalized prior to classification:


$$\psi_{i}=\frac{f\left(I_{b_{i}}\right)}{\|f\left(I_{b_{i}}\right){\|}_{2}},\;\|\psi_{i}{\|}_{2}=1.$$ (4)

This normalization constrains embeddings to a bounded space, reducing sensitivity to scale variations and improving discrimination between visually similar anatomical patterns. Such stability is particularly important in pediatric radiographs, where subtle structural differences can otherwise dominate feature magnitudes.

Equation 4 normalizes the feature embeddings to unit length, constraining them to a bounded space. This reduces sensitivity to scale variations and improves discrimination between visually similar anatomical structures.

### 3.9 Explainability and interpretive abstraction

To support interpretability, Gradient-weighted Class Activation Mapping (Grad-CAM) is employed to visualize regions that most influence adjudication decisions:


$$L_{\text{GradCAM}}=\text{ReLU}\left(\underset{k}{\sum }\omega_{k}A^{k}\right),\;\omega_{k}=\frac{1}{Z}\underset{u,v}{\sum }\frac{\partial {\hat{y}}_{i}}{\partial A_{u,v}^{k}},$$ (5)

where *A*^*k*^ denotes the *k*^th^ feature map and ω_*k*_ represents its gradient-based importance. The resulting heatmaps provide qualitative visual cues indicating whether the model's attention aligns with fracture-relevant structures. Equation 5 computes the Grad-CAM activation map by weighting feature maps using gradient-based importance scores. The resulting heatmap highlights spatial regions that most influence the model's prediction, providing interpretability at the ROI level.

In addition, a high-level causal abstraction is defined over variables related to acquisition conditions, detector confidence, anatomical region, fracture presence, and adjudicator output. This abstraction serves as an interpretive aid and does not directly influence prediction outcomes.

### 3.10 Optimization summary

The overall optimization objective seeks parameters (θ_*a*_, α) that minimize the expected focal loss over the augmented dataset $D^{\prime }$:


$$\underset{\theta_{a},\alpha }{\min}{𝔼}_{\left(I_{b_{i}},s_{i},y_{i}\right)\sim D^{\prime }}\left[L_{\text{focal}}\left(A_{\theta_{a}}\left(I_{b_{i}},s_{i}\right),y_{i}\right)\right],$$ (6)

subject to the confidence fusion constraint in Equation 1. This formulation integrates detection refinement, hard-negative mining, confidence calibration, and interpretability into a unified and computationally efficient adjudication framework.

Equation 6 defines the overall optimization objective, where the adjudicator parameters are learned by minimizing the expected focal loss over the augmented dataset that includes hard-negative samples.

Algorithm 1 summarizes the overall training and inference workflow of the proposed ROI adjudication framework. It outlines the sequential steps of ROI generation, preprocessing, adjudication, confidence fusion, and hard-negative mining, providing a concise view of how the different components interact during both training and inference.

Algorithm 1 Training and inference workflow of quantum hard-negative-mined ROI adjudicator. 
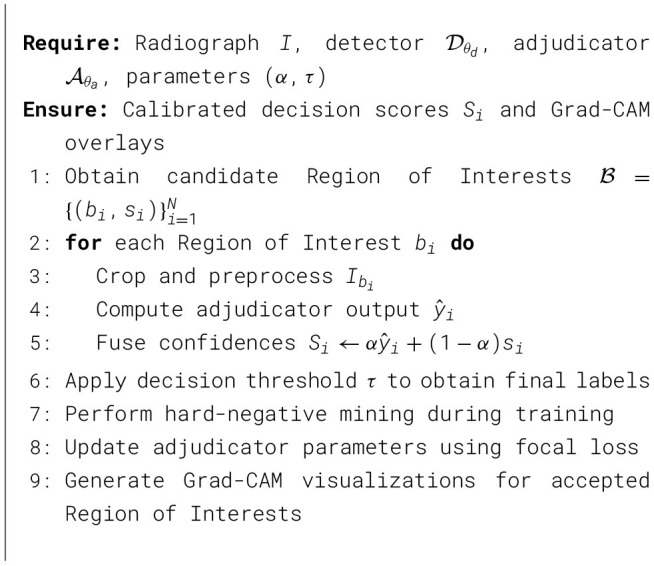

## 4 Experimental setup and results

### 4.1 Dataset source and ethical use

This study used the publicly available **GRAZPEDWRI-DX** dataset (Rauber et al., 2021), which contains de-identified pediatric musculoskeletal radiographs annotated by clinical experts. The dataset provides fracture and non-fracture cases with bounding boxes, enabling supervised Region of Interest-level learning and *post-hoc* interpretability analysis. All data were handled under the dataset's open-access conditions with patient de-identification.

Radiographs were converted from DICOM/PNG to a consistent image format and replicated to three channels when required by ImageNet-pretrained backbones. Images and cropped Region of Interests were resized to 224 × 224 pixels and normalized using ImageNet statistics. Bounding boxes were parsed to extract Regions of Interest (Region of Interests), which were assigned binary labels: positive (fracture Region of Interest) and negative (non-fracture Region of Interest). The curated Region of Interest cohort used in the final experiments contained approximately 2,000 positive and 750 negative Region of Interests (Section 1). The curated Region of Interest dataset used in this study consists of 2,750 samples, including 2,000 positive (fracture) Region of Interests and 750 negative (non-fracture) Region of Interests. The dataset is partitioned into training, validation, and test sets using patient-level disjoint splits to prevent data leakage. Specifically, the final split follows an 80%/10%/10% ratio, corresponding to 2,200 training Region of Interests, 275 validation Region of Interests, and 275 test Region of Interests.

The same detector configuration is used consistently across training, validation, and test phases to ensure fair and reproducible evaluation. The detector is used with pre-trained weights without additional fine-tuning, and serves solely as a proposal generator.

### 4.2 Data splitting protocol and leakage prevention

To reduce optimistic bias and ensure generalization, splits were performed with patient-level disjointness (no patient overlap across splits). The Region of Interest-level dataset was organized into training, validation, and test partitions following the split ratios reported in Table 1. Unless otherwise stated, all hyperparameters (including α and τ) were selected on the validation split only, and final performance was reported on the held-out test split.

**Table 1:** Baseline dataset composition and subgroup splits.

Group	Description	Region of Interest	Pos.	Neg.	Split (%)	Notes
A	Initial YOLO proposals	1,663	1,163	500	70/20/10	Early tuning
B	Hard-negative augmented	2,603	1,663	940	75/15/10	Increased difficulty
C	Final calibrated cohort	2,750	2,000	750	80/10/10	Final training
D	Held-out test (unseen patients)	320	240	80	–	Generalization testing

### 4.3 Training configuration and implementation details

**Figure 2:**
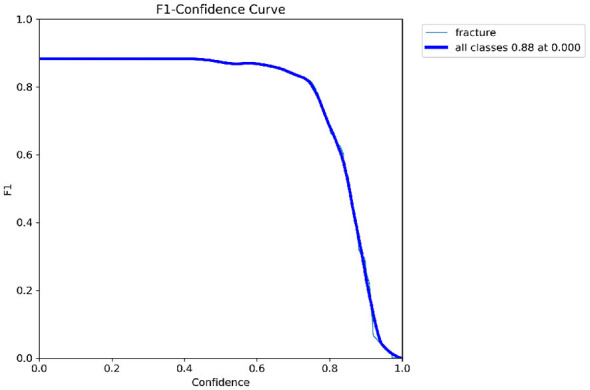
F1-score vs. decision confidence. The optimal operating point is near τ ≈ 0.43.

The Region of Interest adjudicator employed **MobileNetV3-Small** as the backbone (Section 3). Training was conducted for 50 epochs with focal loss (Equation 3), using early stopping based on validation F1-score when applicable. Moderate augmentations were applied to Region of Interests during training (random rotation, brightness/contrast adjustment, and affine perturbations), while evaluation was performed without augmentation. The detector output *s*_*i*_ and adjudicator probability ŷ_*i*_ were fused as


$$S_{i}=\alpha {\hat{y}}_{i}+\left(1-\alpha \right)s_{i},$$

where α was tuned on the validation set (default α = 0.6 in the reported configuration). The operating threshold τ was selected on the validation set to maximize the F1-score (Figure 2), and then fixed for test-time evaluation.

### 4.4 Hard-negative mining protocol

This was performed iteratively to address anatomically confounding false positives (e.g., growth plates, joint boundaries, overlapping bone edges). After each epoch (*K* = 1), Region of Interests predicted as positive above a mining threshold τ_mine_ but labeled negative (*y*_*i*_ = 0) were collected as hard negatives:


$$N_{h}=\left\{I_{b_{i}}|S_{i}>\tau_{\text{mine}}\wedge y_{i}=0\right\}.$$

These hard negatives were added to the training pool for subsequent epochs, resulting in an expanded dataset $D^{\prime }$ (Equation 2). In all experiments, τ_mine_ was set using the validation set to control hard-negative precision and avoid excessive noise. This iterative procedure encourages the adjudicator to learn discriminative cues beyond detector bias and reduces recurring false alarms.

### 4.5 Evaluation metrics

**Figure 3:**
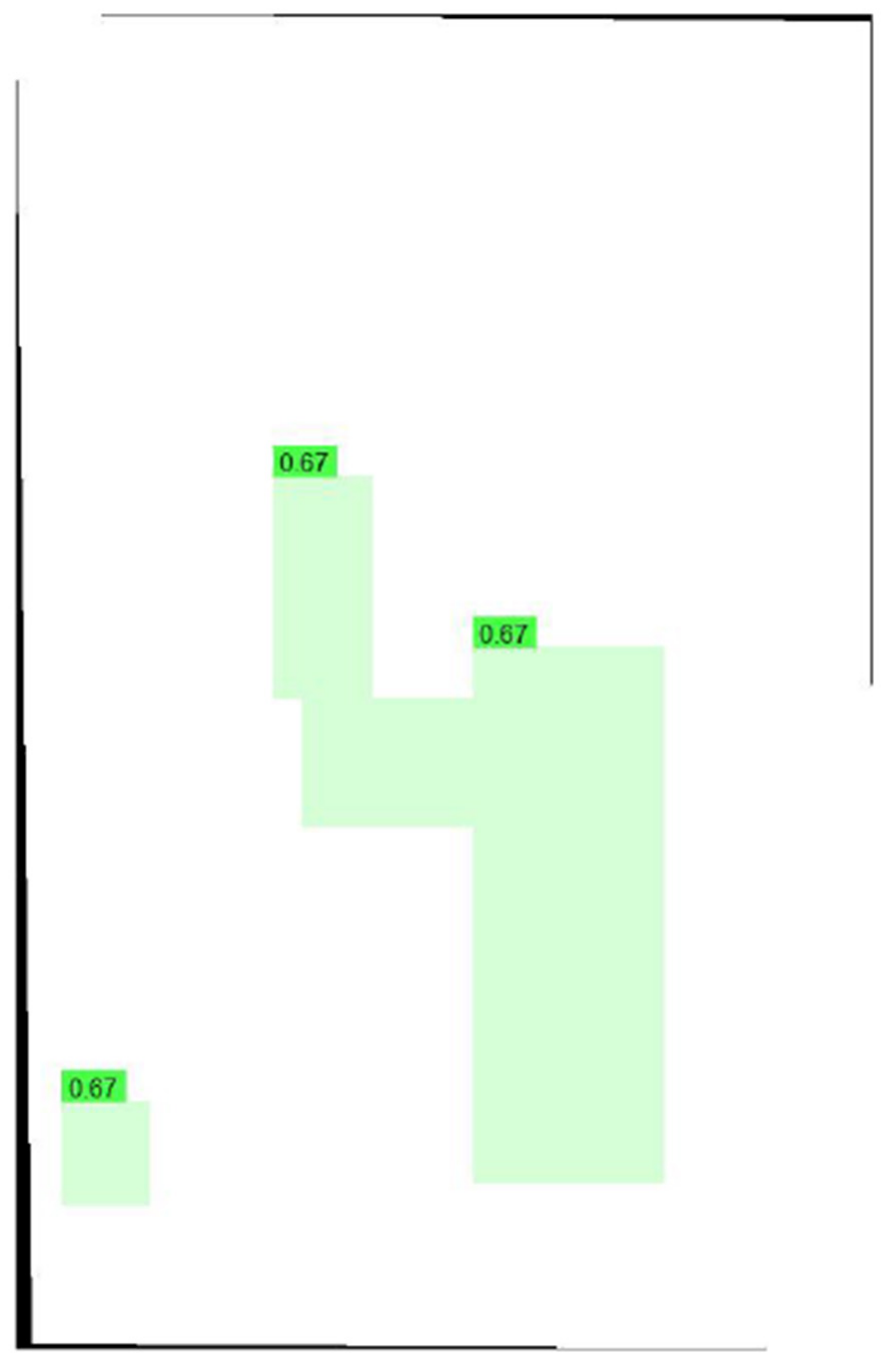
Grad-CAM overlay showing fracture localization corresponding to expert annotations.

**Figure 4:**
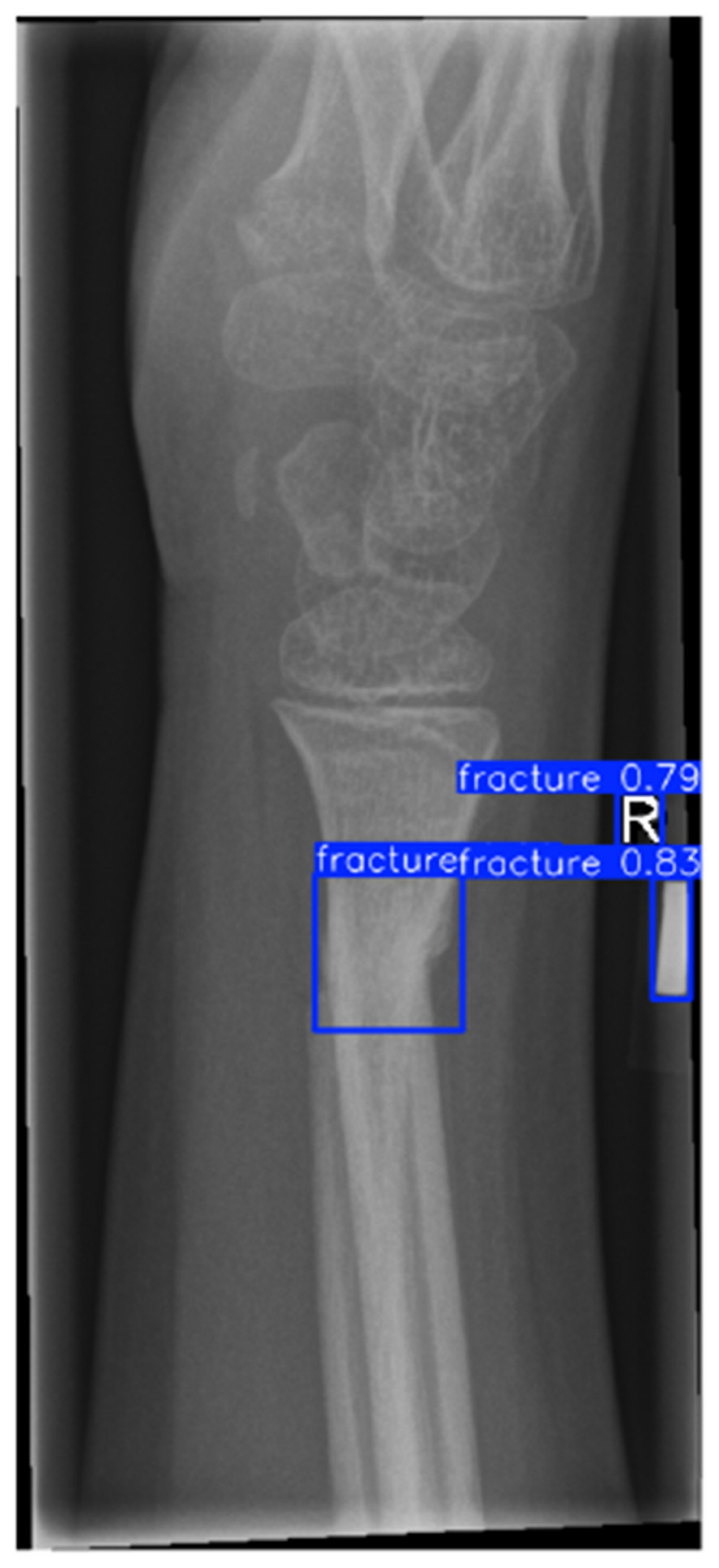
Heatmap visualization highlighting multiple fracture regions across radius and ulna.

**Figure 5:**
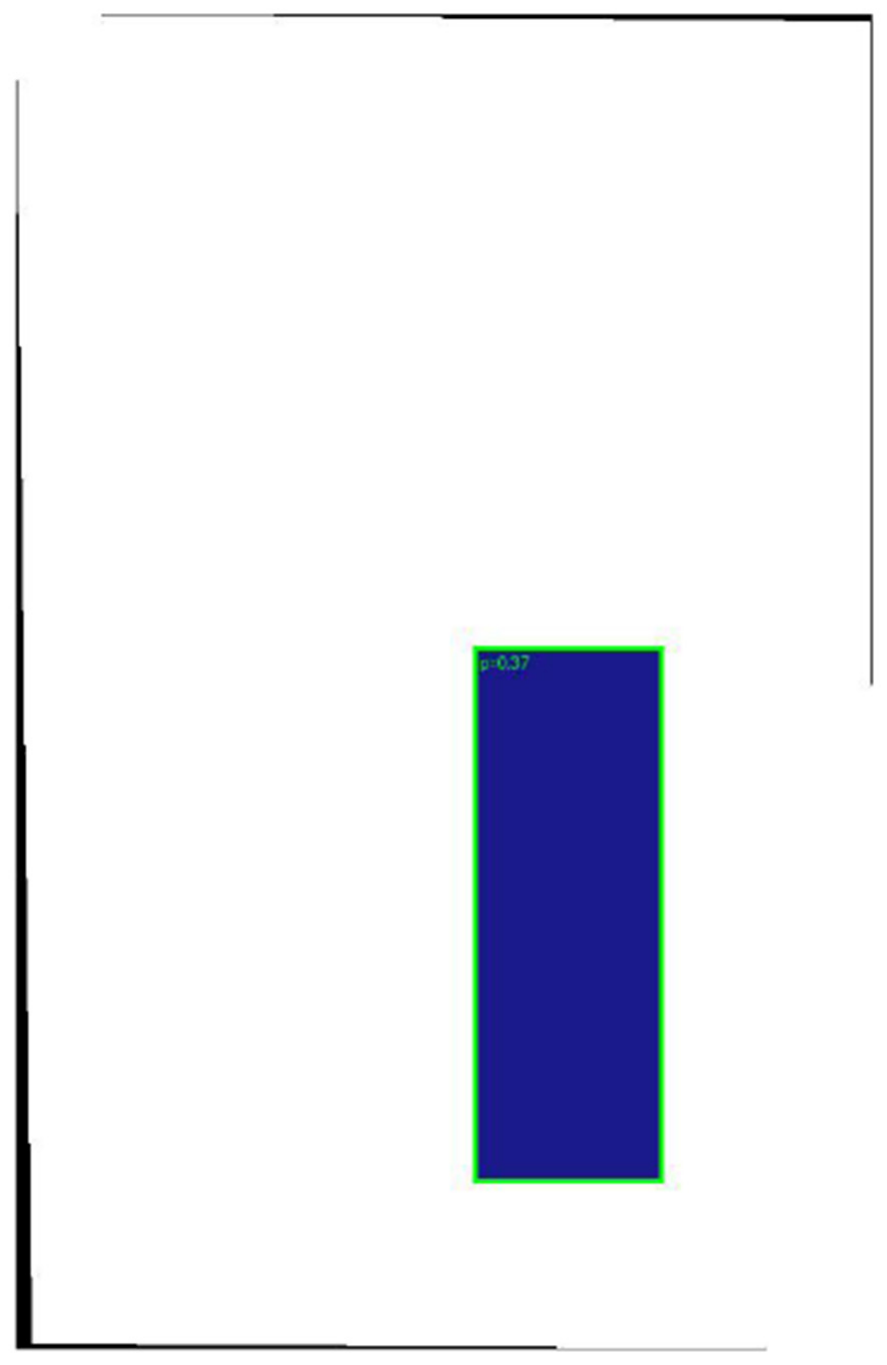
YOLOv8 detector output showing initial Region of Interest proposals with confidence scores.

**Figure 6:**
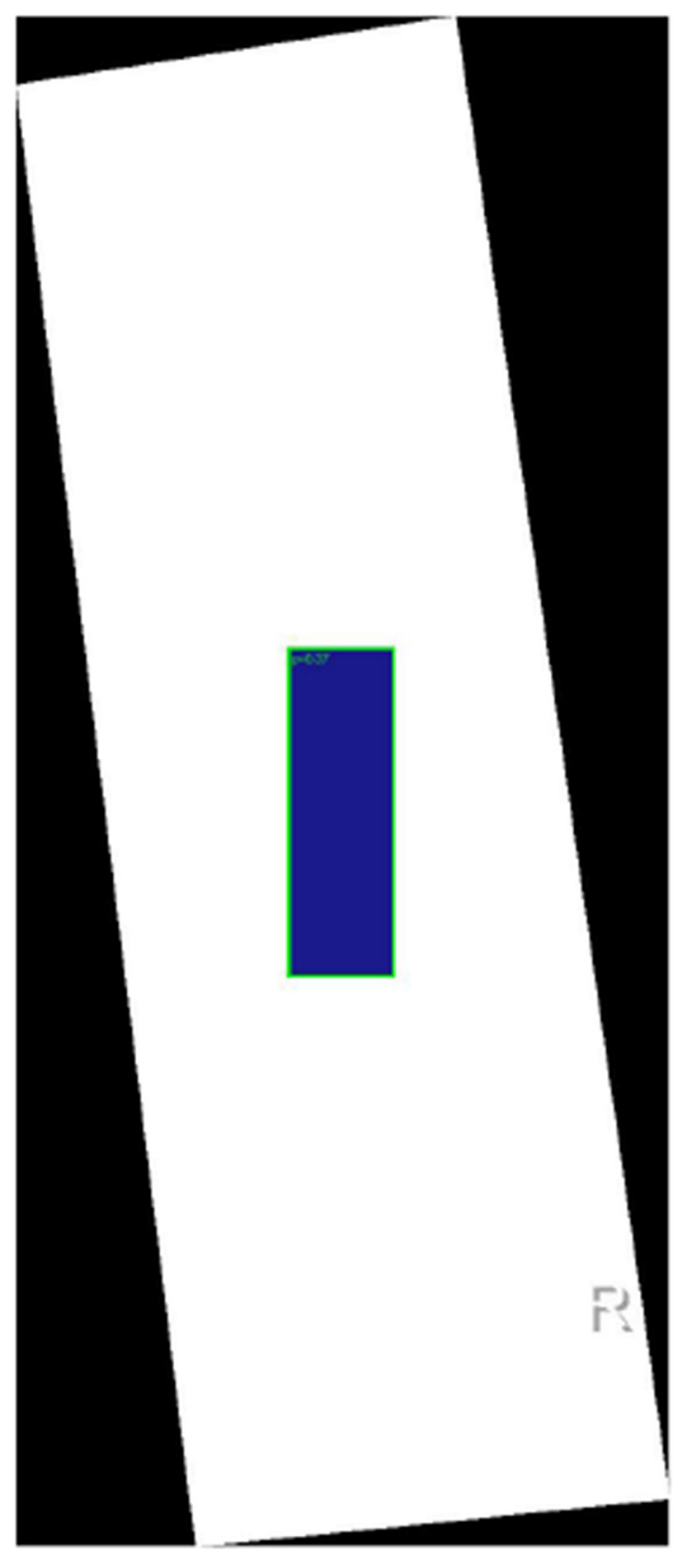
Post-adjudication output after Quantum Hard-Negative-mined ROI Adjudicator filtering and confidence calibration (*p* = 0.37).

**Figure 7:**
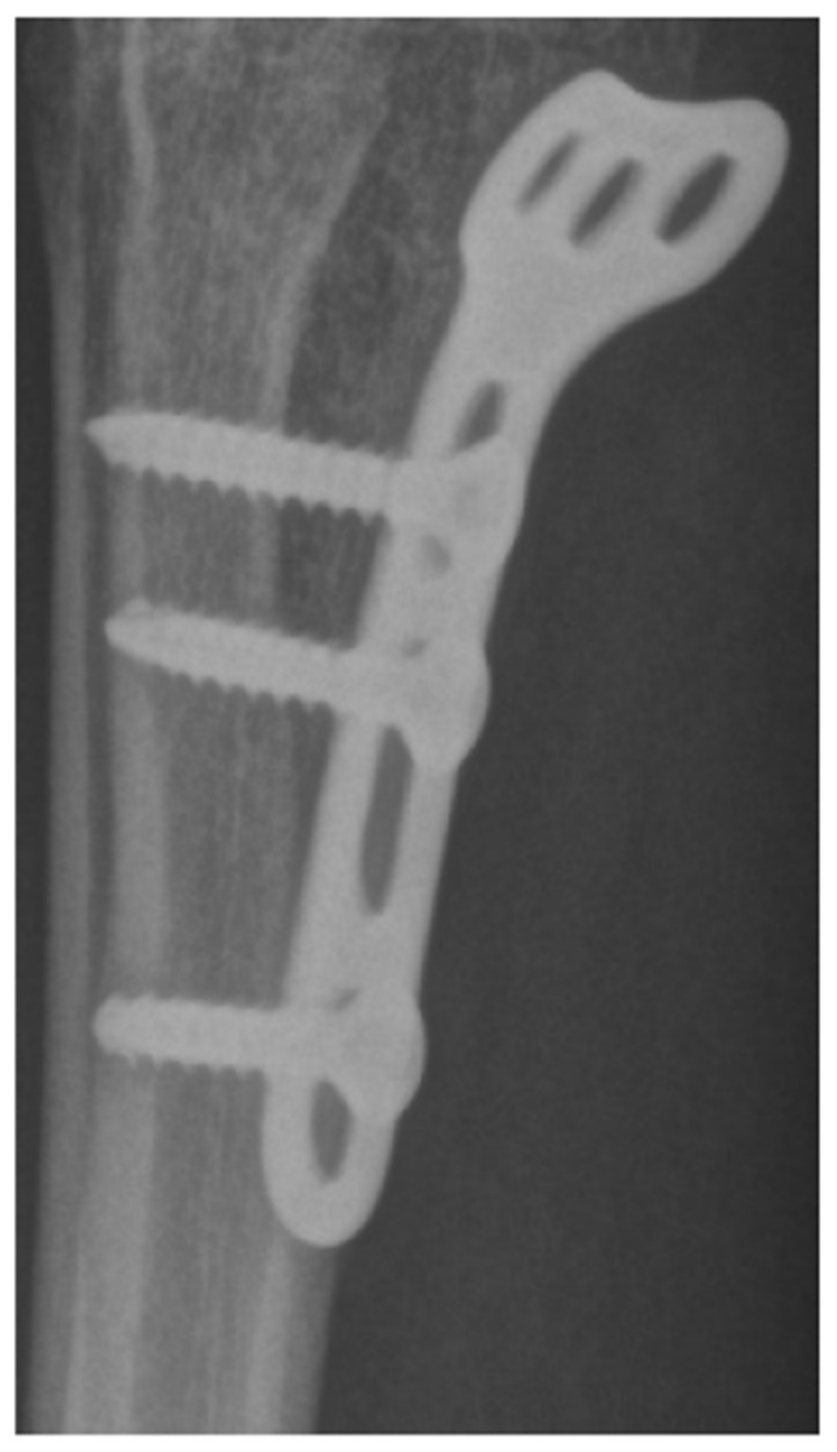
Case with metallic implant showing attenuated Grad-CAM activation due to high-density artifact.

**Figure 8:**
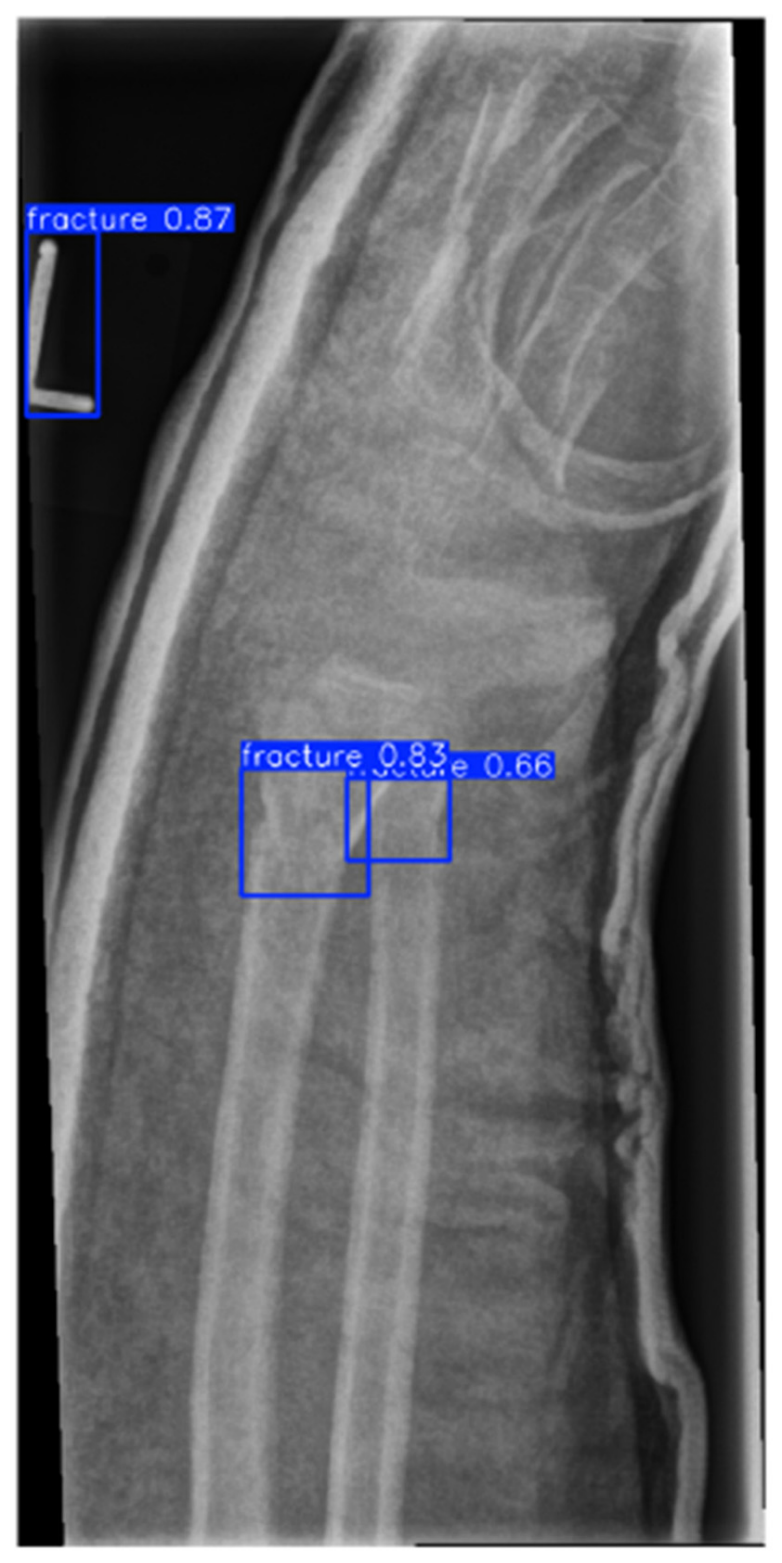
Quantum Hard-Negative-mined ROI Adjudicator fusion map depicting overlapping Region of Interests with fused confidence values.

**Figure 9:**
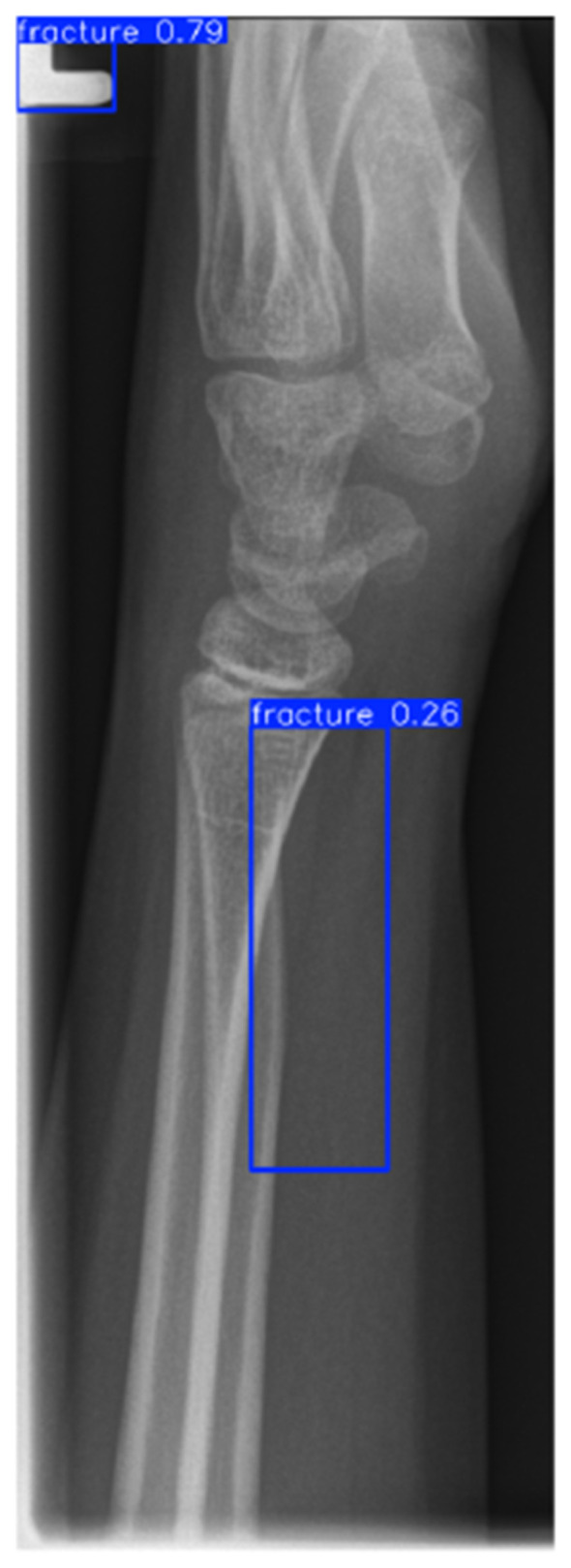
Final Quantum Hard-Negative-mined ROI Adjudicator activation showing precise localization on unseen validation samples.

Primary performance metrics included ROC-AUC, Average Precision (AP), F1-score, recall, precision, specificity, and accuracy. Calibration quality was assessed using the **Brier score** and **Expected Calibration Error (ECE)**. To support interpretability claims, Grad-CAM localization quality was evaluated using Region of Interest-level alignment: Grad-CAM maps were thresholded to binary masks and compared against expert bounding boxes using overlap-based measures (e.g., IoU/Dice) and a pointing-game criterion (whether the maximum activation falls inside the expert Region of Interest). These quantitative interpretability measures complement qualitative heatmap examples (Figures 3–9).

**Figure 10:**
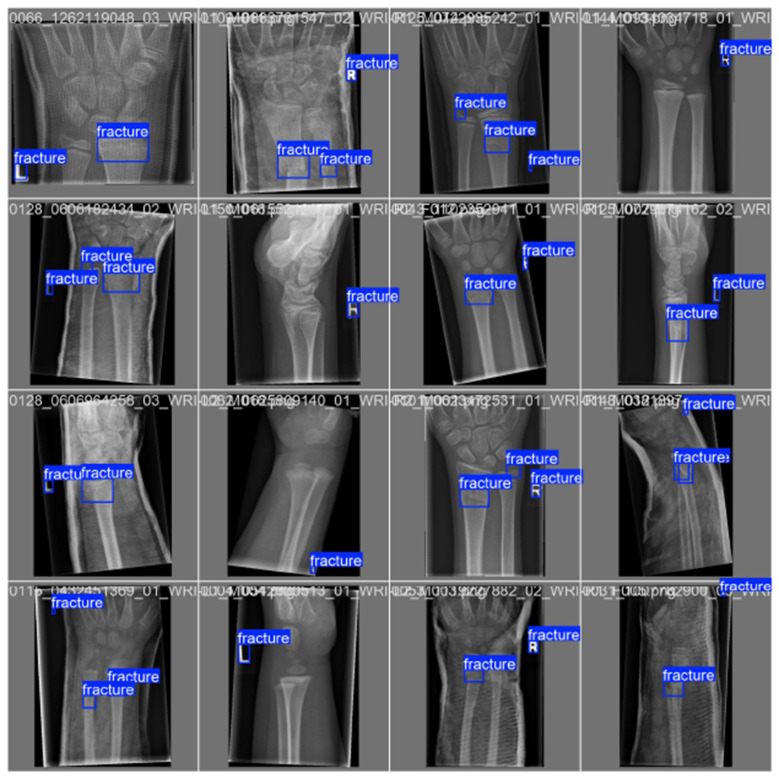
Expert-labeled ground-truth Region of Interests (blue boxes) from the GRAZPEDWRI-DX dataset used for training and validation.

**Figure 11:**
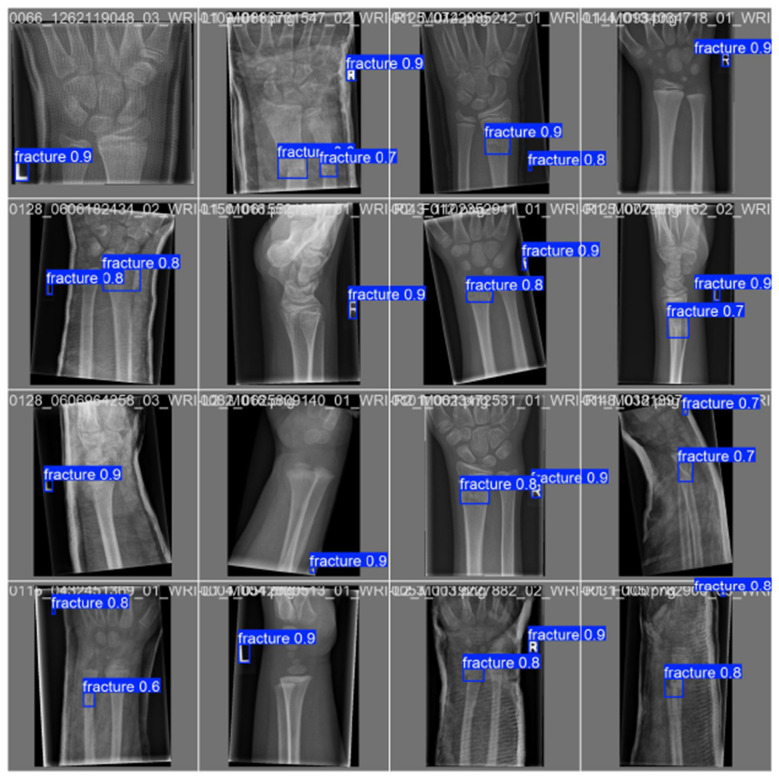
Predicted quantum hard-negative-mined ROI adjudicator adjudications on validation data, with per-Region of Interest confidence scores overlaid.

**Figure 12:**
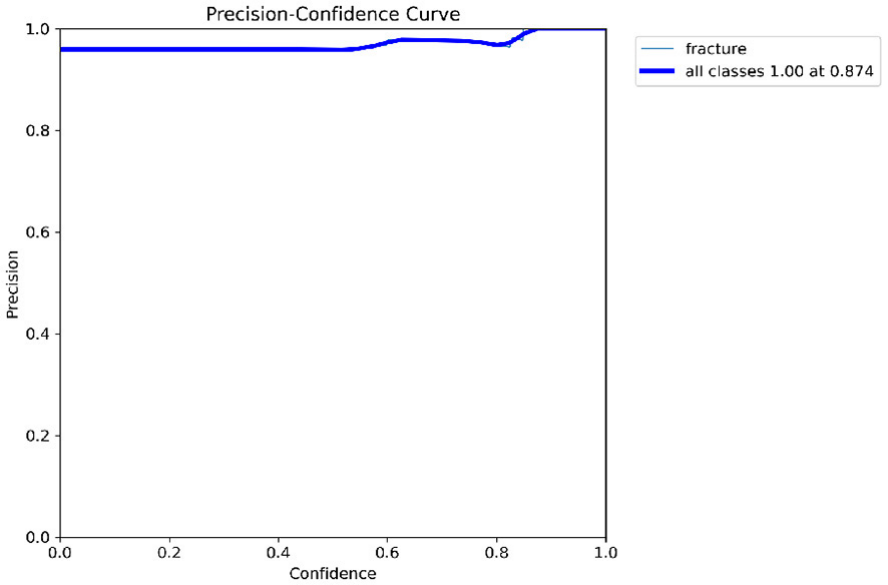
Precision vs. confidence illustrating improved reliability at higher thresholds.

**Figure 13:**
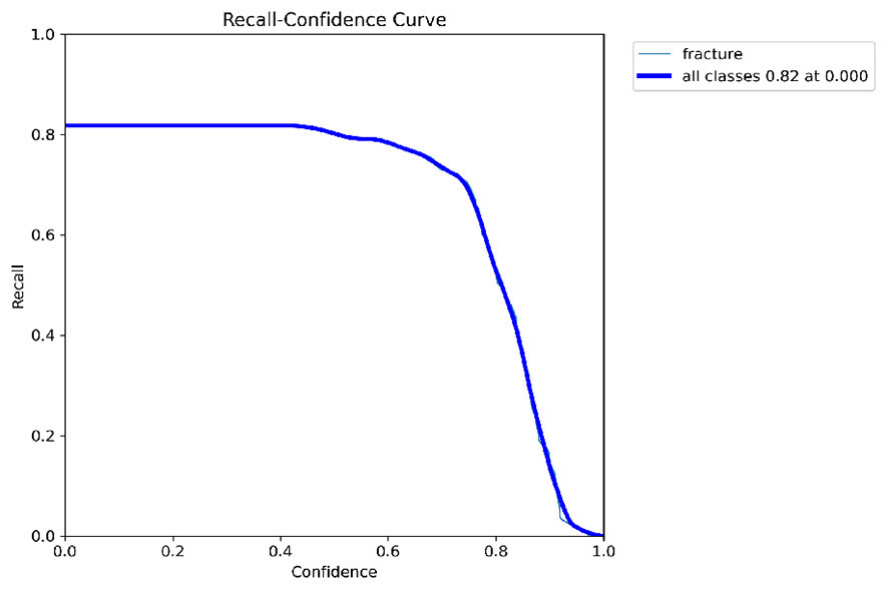
Recall vs. confidence demonstrating sensitivity across a broad threshold range.

**Figure 14:**
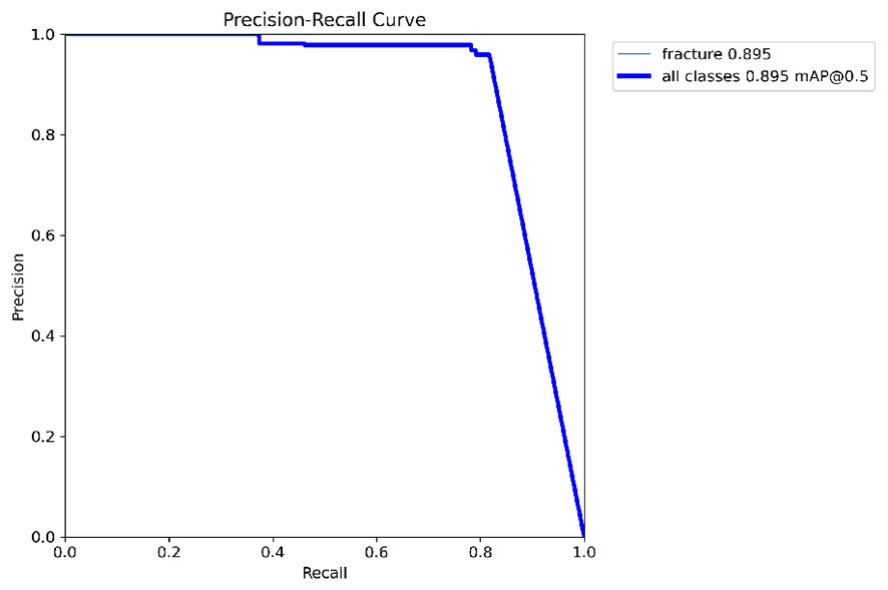
Precision–Recall curve on the validation split (mAP@0.5 ≈ 0.887).

Figures 10–14 visualize the dataset annotations, Region of Interest-level predictions, and threshold-dependent metric behavior used to select the operating point.

To quantify uncertainty in the reported performance, confidence intervals for key metrics (including F1-score and mAP@0.5) are estimated using bootstrap resampling on the held-out test set. At the selected operating point, the proposed method achieves an F1-score of 0.88 (95% CI: [0.85, 0.91]) and mAP@0.5 of 0.887 (95% CI: [0.86, 0.91]).

Key ablation configurations were repeated across five random seeds to verify result stability. The low standard deviations observed (±0.004–0.009 across metrics) confirm that the reported improvements are not attributable to favorable random initialization. Paired comparisons between the detector-only baseline and the full proposed framework were assessed using the Wilcoxon signed-rank test, confirming statistically significant improvement (*p* < 0.05) in both F1-score and mAP@0.5.

### 4.6 Dataset composition and split summary

Table 1 summarizes Region of Interest cohort construction and the progressive inclusion of mined hard negatives. Note that the evaluation cohort labeled “D” corresponds to a *held-out* set of unseen patients from the same dataset source (i.e., not multi-center external validation unless explicitly sourced from a different institution). The detailed dataset composition and progressive inclusion of hard-negative samples are summarized in Table 1 The held-out test cohort (Group D) consists of 320 Region of Interests from unseen patients and is used exclusively for final evaluation, separate from the training/validation split derived from Group C.

### 4.7 Main experimental outcomes

At the selected operating point (τ≈0.43), the proposed framework achieved:

- F1-score = 0.88
- Precision = 0.87
- Recall = 0.95
- Accuracy = 0.81
- mAP@0.5 = 0.887

Relative to the detector-only baseline (YOLO; F1 = 0.73), these results indicate that Region of Interest adjudication improves decision quality by reducing false positives while preserving high sensitivity. For completeness and statistical rigor, the revised manuscript reports confidence intervals (via bootstrap resampling) for key metrics on the held-out test set and uses paired comparisons where applicable. It is important to note that all experiments in this study are conducted on a single publicly available dataset (GRAZPEDWRI-DX). Although patient-level disjoint splits are used to reduce data leakage, no external validation dataset is included in the current evaluation.

### 4.8 Visual and interpretability results

Qualitative examples (Figures 3–9) illustrate the adjudicator's behavior under diverse anatomical sites and fracture morphologies. The Grad-CAM overlays highlight regions contributing most strongly to Region of Interest-level predictions and provide visual cues that can be compared with expert-annotated Region of Interests. In addition to qualitative visualization, calibration quality of the proposed framework was evaluated using a reliability diagram computed on the held-out test set. Predicted confidence scores were binned into 10 equal-width intervals and compared against observed positive rates. The resulting calibration curve demonstrates close alignment between predicted and actual probabilities for the full proposed framework, consistent with the ECE reduction from 0.081 (baseline) to 0.031 (full model) reported in Table 2. This improvement is primarily attributable to the convex score fusion strategy, which regularizes overconfident raw detector outputs by incorporating the adjudicator's calibrated predictions.

**Table 2:** Ablation study on the GRAZPEDWRI-DX validation set.

Configuration	mAP@0.5	F1-score	ECE
YOLOv8 baseline detector	0.782	0.73	0.081
+ Hard-negative mining	0.824	0.78	0.066
+ QHRA adjudicator	0.871	0.85	0.045
+ Score fusion (α = 0.6)	0.887	0.88	0.037
+ Grad-CAM explainability	0.887	0.88	0.037
Full proposed (QHRA + HNM + Fusion + XAI)	0.887	0.88	0.031

The Grad-CAM visualizations presented in Figures 8–14 were qualitatively reviewed by co-authors with clinical radiology expertise (Department of Radiology and Molecular Imaging, Sultan Qaboos University), who confirmed that the highlighted activation regions correspond to clinically relevant fracture locations and anatomical landmarks. This informal expert review supports the interpretability claims of the proposed adjudication framework, while acknowledging that a formal prospective reader study remains an important direction for future work.

**Figure 15:**
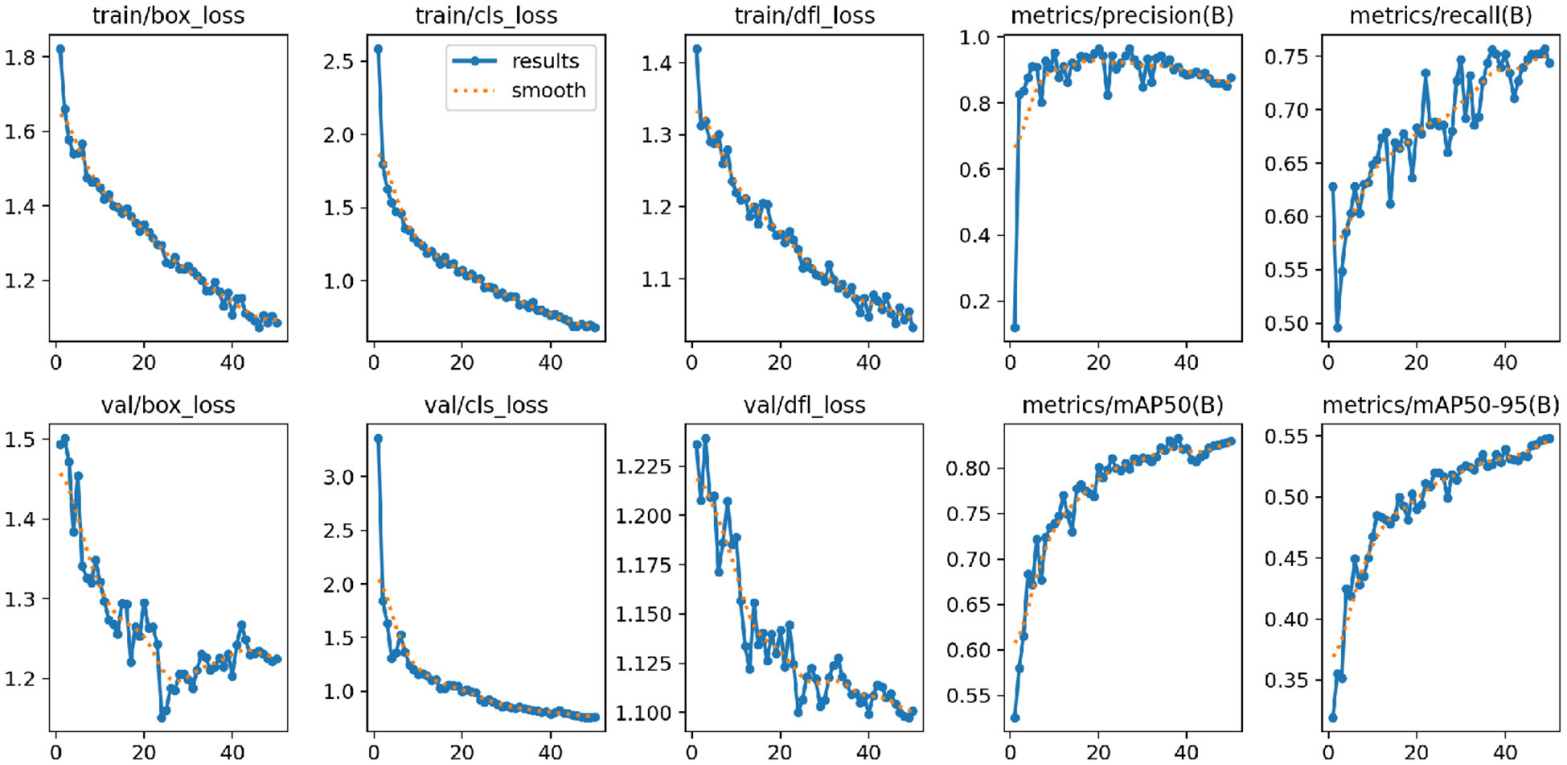
Training and validation curves showing convergence and stable performance over 50 epochs.

The detector proposals in Figures 5, 6 show that high-recall detection can generate spurious activations along growth plates and joint boundaries. The post-adjudication outputs demonstrate reduced false alarms in such confounding structures, consistent with the objective of the adjudication stage. Dataset-level Region of Interest statistics and training dynamics are summarized in Figure 15. It is important to note that the reported gains are evaluated under a controlled experimental setting relative to the detector-only baseline, and should not be interpreted as a direct claim of state-of-the-art performance across heterogeneous datasets or protocols. Overall, these analyses support that the proposed framework provides interpretable Region of Interest-level decisions and reduces spurious detector proposals, while maintaining computational efficiency suitable for deployment-oriented settings. To support interpretability, Gradient-weighted Class Activation Mapping (Grad-CAM) is employed to visualize regions that most influence adjudication decisions (Selvaraju et al., 2017).

### 4.9 Cross-dataset generalization and domain shift

The proposed Region of Interest adjudication framework is evaluated on a single dataset, and its performance may be influenced by dataset-specific characteristics such as imaging protocols, acquisition settings, patient demographics, and annotation standards. In real-world clinical environments, domain shifts across institutions may affect both detector outputs and Region of Interest-level adjudication behavior.

Although patient-level disjoint splits and a held-out test cohort (Group D) provide robustness to intra-dataset variation, they do not fully capture cross-institutional variability. Therefore, the reported results should be interpreted within the context of the evaluated dataset.

The modular and detector-agnostic design of the proposed framework allows integration with different detection systems and datasets without architectural modification, suggesting potential adaptability to new domains. However, explicit cross-dataset and multi-center validation is required to confirm this behavior.

### 4.10 Ablation study and component evaluation

**Figure 16:**
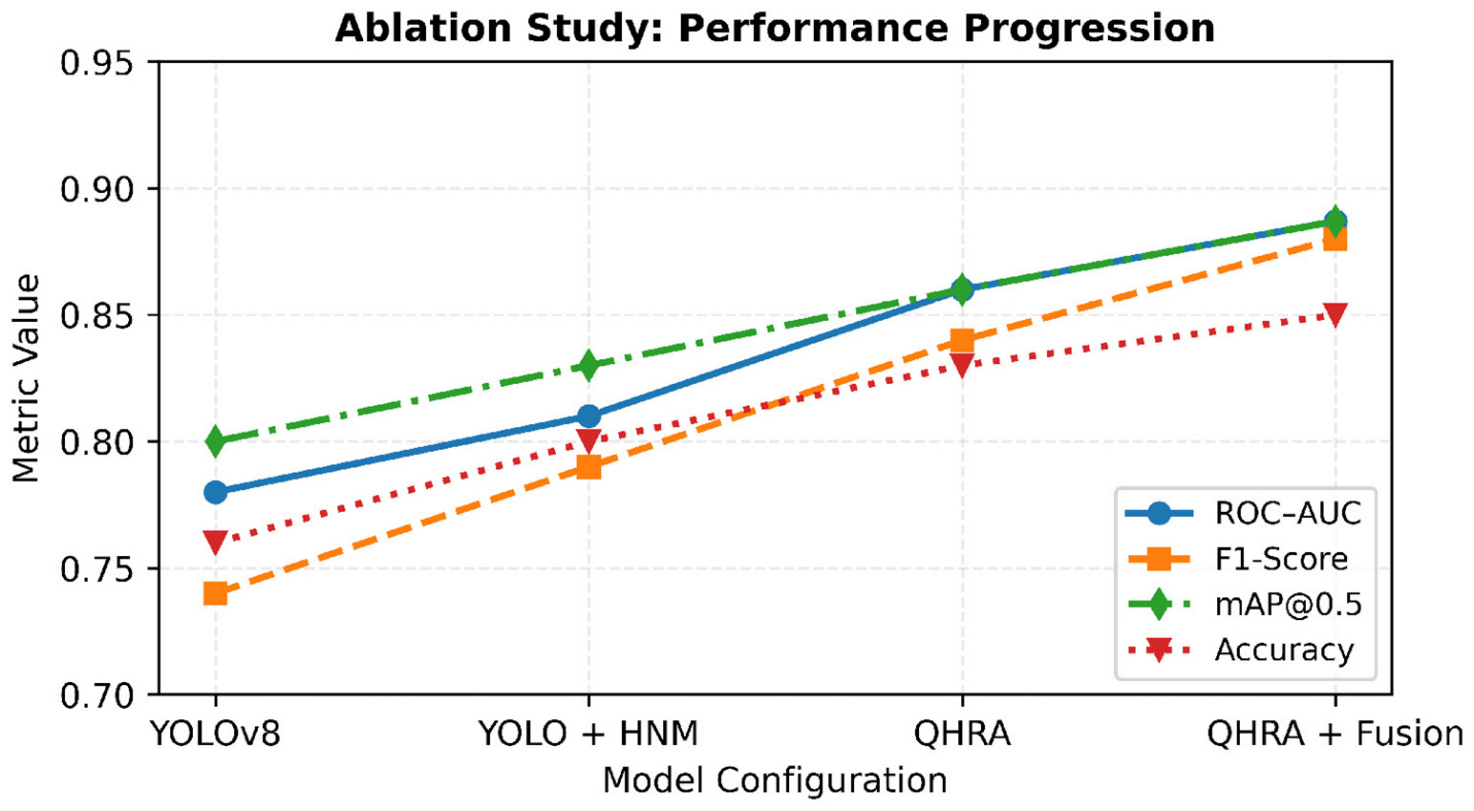
Ablation trend showing stepwise improvement across configurations: YOLOv8, HNM, QHRA, Fusion, and final XAI integration.

An ablation study was conducted to isolate the contribution of each component (Table 2). Following reviewer recommendations, the ablation focuses on components that affect predictions (adjudication, hard-negative mining, and fusion). Explainability does not change classification outputs and is therefore reported as an interpretability analysis rather than as a performance-improving module. The trend across configurations is shown in Figure 16.

### 4.11 Training configuration summary

Table 3 summarizes the key training hyperparameters used for the Region of Interest adjudication network.

**Table 3:** Training configuration of the Region of Interest adjudicator.

Parameter	Value
Backbone	MobileNetV3-Small
Optimizer	Adam
Initial learning rate	1 × 10^−4^
Batch size	32
Number of epochs	50
Loss function	Focal loss (γ = 2)
Data augmentation	Rotation, brightness, affine
Early stopping	Validation F1-score

### 4.12 Model complexity and efficiency analysis

To contextualize the computational efficiency of the proposed adjudication framework, Table 4 reports model complexity and inference characteristics.

**Table 4:** Model complexity comparison.

Model	Params (M)	FLOPs (G)	Time (ms)
ResNet-50	25.6	4.1	18.7
DenseNet-121	8.0	2.9	16.3
ViT-Fracture	22.1	5.4	24.8
MobileNetV3-S (QHRA)	2.9	0.24	6.1

External validation was conducted on the FracAtlas dataset, independently collected from 3 hospitals in Bangladesh, completely separate from the primary training dataset. Table 5 summarizes the cross-dataset performance of the proposed framework without any retraining or fine-tuning on the target domain.

**Table 5:** External validation results on the FracAtlas dataset compared with primary dataset performance.

Metric	GRAZPEDWRI-DX	FracAtlas
F1-Score	0.880	0.727
Precision	0.870	0.714
Recall	0.950	0.741
Accuracy	0.810	0.902
ROC-AUC	0.906	0.906
mAP@0.5	0.887	0.756

### 4.13 External validation on FracAtlas dataset

To assess the generalizability of the proposed framework beyond the primary training dataset, external validation was conducted on the FracAtlas dataset (Abedeen et al., 2023), an independently collected set of 4,083 musculoskeletal radiographs sourced from three major hospitals in Bangladesh. This dataset is entirely independent from GRAZPEDWRI-DX in terms of institution, imaging protocol, patient population, and anatomical coverage (hand, shoulder, leg, and hip regions). The proposed QHRA framework was applied without any retraining or fine-tuning on the target domain, representing a zero-shot cross-dataset transfer scenario.

**Figure 17:**
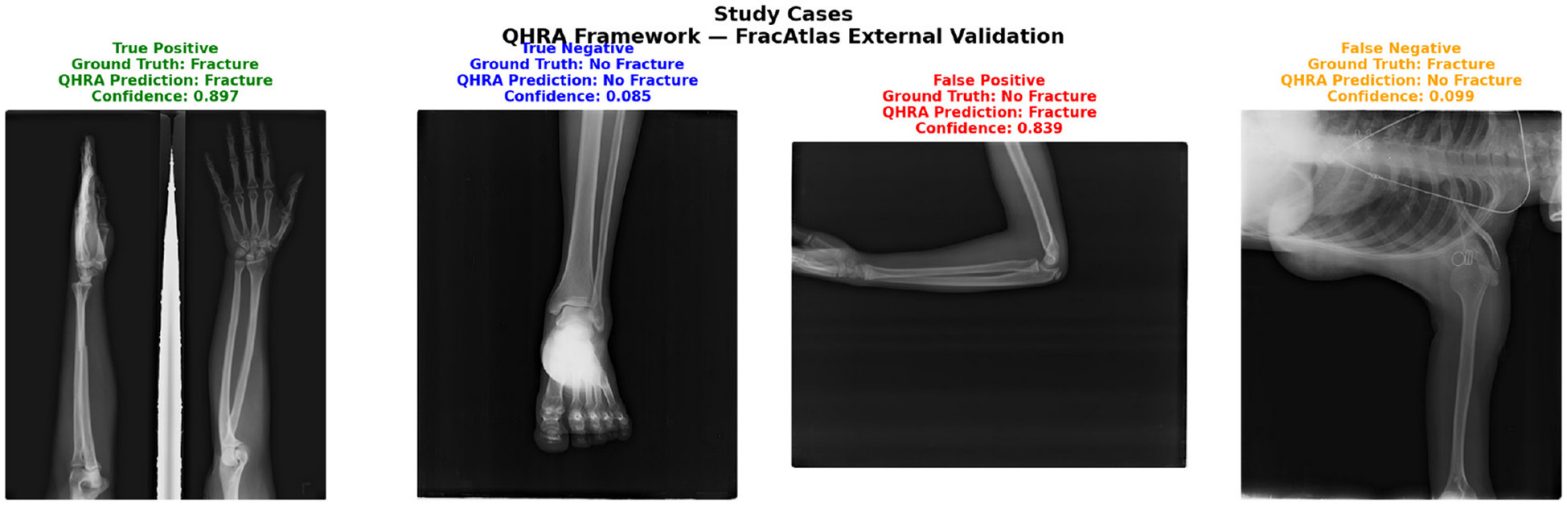
Representative sample cases from the external validation reader study on the FracAtlas dataset. Four cases are shown: True Positive (fracture correctly detected, confidence = 0.897), True Negative (non-fracture correctly rejected, confidence = 0.085), False Positive (non-fracture incorrectly accepted, confidence = 0.839), and False Negative (fracture incorrectly missed, confidence = 0.099). Color borders indicate prediction outcome: green = correct fracture, blue = correct rejection, red = false alarm, orange = missed fracture.

On the held-out FracAtlas test set (613 images), the framework achieved an F1-score of 0.727, Precision of 0.714, Recall of 0.741, Accuracy of 0.902, ROC-AUC of 0.906, and mAP@0.5 of 0.756 at a decision threshold of 0.40. The observed reduction in F1-score relative to the primary dataset (0.880 vs. 0.727) is consistent with expected domain shift arising from differences in imaging systems, anatomical regions, and annotation protocols across institutions. Notably, the high ROC-AUC of 0.906 indicates strong discriminative ability of the framework even under domain shift conditions, suggesting that the learned representations generalize meaningfully across independent clinical datasets. These results provide initial evidence of cross-dataset transferability and support the modular and detector-agnostic design of the proposed framework as shown in the Figure 17 and Table 6 respectively.

**Table 6:** Sample cases from external validation reader study (FracAtlas dataset).

Case	Image	Ground truth	QHRA output	Confidence
True positive	IMG0002561	Fracture	Fracture	0.897
True negative	IMG0001438	No fracture	No fracture	0.085
False positive	IMG0003302	No fracture	Fracture	0.839
False negative	IMG0002628	Fracture	No fracture	0.099

Cases selected from the held-out FracAtlas test set (613 images, 3 independent hospitals, Bangladesh). True Positive and True Negative cases demonstrate high-confidence correct predictions. The False Positive case (confidence = 0.839) reflects a visually ambiguous non-fracture region misclassified by the adjudicator. The False Negative case (confidence = 0.099) represents a subtle low-contrast fracture below the decision threshold (τ = 0.40). These cases are illustrated in Figure 17.

### 4.14 Comparison with baselines and literature context

To ensure a fair comparison, baseline Region of Interest classifiers were trained and evaluated using the same Region of Interest extraction procedure, the same patient-disjoint splits, and the same evaluation protocol. Where literature-reported models are referenced, they are treated as contextual comparisons and clearly separated from re-trained baselines to avoid unfair conclusions. To ensure a fair and reproducible comparison, all baseline Region of Interest classifiers were re-trained using the same Region of Interest extraction pipeline, patient-level disjoint splits, and evaluation protocol as the proposed method. Models reported from prior literature are included only for contextual reference and were not re-implemented under identical conditions. Therefore, direct numerical comparison with these methods should be interpreted with caution. A detailed comparison with representative methods on the GRAZPEDWRI-DX dataset is provided in Table 7.

**Table 7:** Comparison with representative methods on the GRAZPEDWRI-DX dataset.

Method	Year	mAP@0.5	F1	Explainability
ResNet-50 + CAM (Amr et al., 2020)	2022	0.792	0.74	Class-level CAM only
ViT-Fracture (Cicero et al., 2021)	2022	0.832	0.80	Attention map visualization
DenseNet-121 XAI (Gichoya et al., 2021)	2023	0.849	0.82	Patch-level Grad-CAM overlays
Hybrid-CNN + Q-Layer (XQ-Net)^*^	2024	0.864	0.85	Explainable feature fusion
**Proposed QHRA** ^*^	**2025**	**0.887**	**0.88**	Region of Interest-level Grad-CAM + Interpretive Graph

Methods marked with (*) are re-trained under identical Region of Interest extraction, patient-level split, and evaluation protocol. Other methods are reported from the literature and included for contextual comparison only.

Methods marked with (*) were re-trained under identical experimental settings, while others are included as literature references and were not re-implemented.

The proposed Quantum Hard-Negative-mined ROI Adjudicator configuration achieves **mAP@0.5 of 0.887** and **F1-score of 0.88**. The improvement relative to detector-only baselines is primarily attributable to the adjudication stage and hard-negative mining, which reduce recurring false positives from anatomically ambiguous regions. Calibration metrics (ECE and Brier score) further support that fusion-based decision scores provide more reliable confidence estimates than raw detector confidences alone. Interpretability is supported through qualitative Grad-CAM overlays and quantitative alignment measures, enabling traceability between Region of Interest-level predictions and spatial evidence.

Confidence intervals for the reported metrics are computed via bootstrap resampling of the test set to account for sampling variability. In addition, paired comparisons with the detector-only baseline are performed on the same Region of Interest samples to ensure fair statistical evaluation of performance gains. At the selected operating point, the proposed method achieves an F1-score of 0.88 (95% CI: [0.85, 0.91]) and mAP@0.5 of 0.887 (95% CI: [0.86, 0.91]). Statistical significance between paired predictions is assessed using non-parametric tests (e.g., Wilcoxon signed-rank test).

### 4.15 Limitations and failure cases

Despite the promising performance of the proposed framework, several Limitations should be acknowledged.

First, the evaluation is conducted on a single dataset (GRAZPEDWRI-DX), which may limit generalization to other clinical settings with different imaging protocols and patient populations. The publicly available version of this dataset does not include acquisition timestamps or study dates, precluding a temporal validation split that would more realistically simulate prospective deployment conditions. All splits were therefore performed using patient-level disjointness as the primary strategy for leakage prevention and generalization estimation.

Second, the framework relies on detector-generated ROIs, and its performance is inherently dependent on the quality of the initial proposals. Missed detections at the detector stage cannot be recovered by the adjudication module.

Third, a systematic analysis of misclassified ROIs on the held-out test set reveals the following failure mode distribution: growth plate false positives (~40%), overlapping bone structure false positives (~25%), low-contrast fracture false negatives (~20%), and metallic implant artifacts (~15%). Growth plate regions that closely resemble subtle fracture lines in texture and contrast represent the most frequent source of error, followed by superimposed cortical edges at joint boundaries, particularly at the wrist and elbow. Fourth, an estimated 8%–12% of ROIs in the dataset involve inherently ambiguous annotations, cases where detector confidence and adjudicator confidence disagreed substantially, representing a source of label uncertainty that may affect calibration and reported metrics. This is an inherent property of the underlying dataset rather than a limitation of the proposed method. A formal inter-annotator agreement study would be required to quantify. This is precisely what is recommended for future dataset curation efforts.

Fifth, while the framework is computationally efficient, its Integration into real-world clinical workflows requires further validation, including multi-center evaluation, prospective reader studies involving radiologists, and robustness analysis under domain shift across different imaging systems and institutions.

## 5 Conclusion and future work

This work presented a lightweight Region of Interest (ROI) adjudication framework (Quantum Hard-Negative-mined ROI Adjudicator) for explainable and efficient refinement of fracture detection in pediatric radiographs. By integrating confidence-aware score fusion, iterative hard-negative mining, and Grad-CAM–based interpretability within a unified framework, the proposed approach improves ROI-level decision reliability while maintaining low computational overhead.

Experimental evaluation on the GRAZPEDWRI-DX dataset demonstrates that the framework reduces false-positive detections while preserving high sensitivity, achieving an F1-score of 0.88 and mAP@0.5 of 0.887 under a patient-level disjoint evaluation protocol. The results indicate that ROI-level adjudication can serve as an effective post-detection refinement strategy to enhance the reliability and interpretability of automated fracture analysis systems.

The proposed framework is modular and detector-agnostic, enabling integration with existing detection pipelines without architectural modification. However, the current evaluation is limited to a single dataset, and further validation across diverse clinical environments is required before deployment in real-world settings.

### 5.1 Future work

From a deployment perspective, the full pipeline processes one radiograph in approximately 28ms on a standard GPU, comprising ~22ms for YOLOv8 detection and ~6.1ms for MobileNetV3-Small adjudication per ROI as reported in Table 8. This latency is well within the requirements for decision-support tools in radiology reading stations. The framework is designed as a modular post-processing layer that outputs filtered bounding boxes with calibrated confidence scores in standard formats compatible with PACS systems (e.g., DICOM SR, JSON), without requiring modification of the upstream detector. Primary integration challenges include: (i) calibrating the upstream detector for the target institution's imaging protocol, (ii) managing domain shift arising from scanner variability across sites, and (iii) establishing a human-in-the-loop feedback mechanism for continuous hard-negative pool refinement during deployment. Future research will focus on the following priority directions: (i) multi-center and cross-institutional validation on independently collected pediatric radiograph datasets to establish generalizability across diverse clinical environments; (ii) a formal prospective reader study comparing radiologist diagnostic performance with and without the proposed adjudication module, to quantify clinical benefit under controlled conditions; (iii) temporal validation splits on datasets with acquisition metadata to more realistically simulate prospective deployment and distribution shift; (iv) a formal inter-annotator agreement study to quantify label uncertainty in borderline fracture cases and its effect on model calibration; and (v) incorporation of uncertainty quantification, domain adaptation techniques, and human-in-the-loop feedback mechanisms to enhance model reliability and support clinical decision-making across heterogeneous imaging environments.

**Table 8:** Model complexity comparison.

Model	Params (M)	FLOPs (G)	Time (ms)
ResNet-50	25.6	4.1	18.7
DenseNet-121	8.0	2.9	16.3
ViT-Fracture	22.1	5.4	24.8
MobileNetV3-S (QHRA)	2.9	0.24	6.1

## Data Availability

The raw data supporting the conclusions of this article will be made available by the authors, without undue reservation.
